# Involvement of community pharmacists in continuing professional development (CPD): a baseline survey in Gondar, Northwest Ethiopia

**DOI:** 10.1186/s12992-018-0334-0

**Published:** 2018-02-01

**Authors:** Dessalegn Asmelashe Gelayee, Gashaw Binega Mekonnen, Mequanent Kassa Birarra

**Affiliations:** 1 Department of Pharmacology, College of Medicine and Health Sciences, University of Gondar, P.O.Box 196, Gondar, Ethiopia; 2 Department of Clinical Pharmacy, College of Medicine and Health Sciences, University of Gondar, P.O.Box 196, Gondar, Ethiopia

**Keywords:** Community pharmacist, Continuing professional development (CPD), Ethiopia

## Abstract

**Background:**

Health care professionals have been striving to maintain their competence to deliver the best quality of service. This study intended to determine involvement in continuing professional development of community pharmacists in Gondar, Northwest Ethiopia.

**Methods:**

About 46 community pharmacists, each from a different setting, were interviewed using structured questionnaire. Data were analyzed using Pearson’s Chi-square test of independence and Mann-Whitney U test with *p* < 0.05 taken as statistically significant.

**Results:**

The majority (*n* = 26, 56.5%) reported of being unaware of the CPD concept. The mean hour spent per week on CPD is 4.1 ± 4.0. Most (*n* = 34, 73.9%) were engaged in self directed learning and expressed an interest to be more involved in CPD activities (*N* = 39, 84.8%). Interactive workshops were the most preferred modality. However they seek further support in the process of identifying learning needs (*N* = 34, 73.9%). The main barriers for CPD engagement include lack of (*N* = 36, 78.3%) and inaccessibility (N = 34, 73.9%) of CPD opportunities as well as time shortage (*N* = 33, 71.7%).

**Conclusions:**

The community pharmacists in Gondar, Northwest Ethiopia lack awareness of CPD concept but engaged in various types of CPD activities. They demonstrated good attitude and seek more support. The main barrier was lack of opportunities related to CPD.

## Background

Health care professionals have been striving to maintain their competence to deliver the best quality of service. All are ethically required to maintain competency throughout their career [1].

The exponential progress in technology, diagnostic tools and treatment methods, as well as changing population demographics and disease burden, makes updating and maintaining the knowledge and skills of health workers throughout their professional life more important than ever [2]. Pharmacists’ professional role for instance has continued its expansion and thus the advancing science and patient safety issues require pharmacists to maintain their competency. In this regard, implementing Continuing Professional Development (CPD) programs will be very important. The International Pharmaceutical Federation (FIP) has adopted the concept of CPD in 2002 and defined CPD as the responsibility of an individual pharmacist for systematic maintenance, development and broadening of knowledge, skills and attitudes to ensure continuing competence as a professional throughout their career [1]. In this process the individual practitioner determines his own learning needs, makes plans to meet those objectives, executes those plans, and finally evaluates whether the actions were successful [3]. Continuing education (CE) can be seen as one part of the CPD process, encompassing such traditional teaching methods as lectures, workshops, and distance learning courses [3]. The major advantage of CPD over CE is that for CPD, learning can be linked to the workplace as it is intended to be more experiential and informal. Many of the daily activities such as analyzing critical incidents at work and structured reading can constitute as CPD if recorded correctly [4].

Majority of health professionals are accustomed to participating in continuing education (CE) in the form of conferences and meetings. However, health professionals are increasingly expected to be more self-directed in their learning [5]. CE has proven to be an insufficient method of changing pharmacists’ behavior [3]. A study comparing the impact of continuing professional development versus traditional continuing pharmacy education on pharmacy practice identified a better out come for CPD [6].

Increasingly, more countries worldwide are implementing CE program for community pharmacists as obligatory lifelong learning programs in an attempt to improve clinical community pharmacy services [7].

Ethiopia is setting up a CPD system and to facilitate it, CPD Guideline for Health Professionals is introduced in 2013 [8]. In the nation there is a shift in trend of pharmacy practice towards patient-focused one [9]. Such shift is known to create a need to develop and maintain expertise and competence in new areas, including pharmacotherapy and interpersonal communication among others [7].

The objectives of this study were to identify the pattern of CPD practice, attitude, preferences and barriers to engagement on CPD of community pharmacists in Gondar town, Northwest Ethiopia. It can support in designing and implementing CPD approaches in the context of community pharmacy practice.

## Methods

A cross-sectional study was conducted among pharmacists working in the community pharmacies in Gondar Town, Northwest Ethiopia in November 2016. About 53 medication retail outlets (19 pharmacies and 34 drug stores) were registered in the town in 2014 [10]. In the present study community pharmacist refers to at least diploma holders in Pharmacy education and community pharmacy refers both drug stores and pharmacies. Data were collected using a structured interviewing questionnaire on 46 pharmacists who gave their consent for participation in the study. The data collection instrument was developed based on literature reviews and consisted of closed and 4 point Likert-type scale questions (strongly disagree, disagree, agree, strongly agree) on socio-demographic characteristics, awareness on CPD concept, attitude and practice related to CPD, type of CPD preferences, average time spent on CPD per week, if received any support on CPD, as well as barriers for engaging in CPD. The questionnaire was sent to four senior pharmacists who are academicians and researchers for face validity and approval was obtained. It was pretested on 5 pharmacy technicians working as part time in private pharmacies and some modifications were made. The collected data were cleared and entered into and analyzed by using the statistical package for social sciences (SPSS) version 20.0 for windows (SPSS Inc., Chicago, Illinois). The reliability assessment of the different sub-components of the questionnaire post data collection revealed a Cronbach’s alpha value of 0.867 for attitude on CPD (6 items), 0.725 for CPD practice (7 items) and 0.744 for barriers in CPD engagement (10 items). The results were described in terms of frequencies, percentages and means. The relationships among variables were analyzed by using Mann-Whitney U test and Pearson’s chi-square test of independence with a *p*-value <0.05 considered statistically significant. An ethical clearance was taken from the Ethical Review Committee of School of Pharmacy, University of Gondar and verbal consent was obtained from the participants.

## Results

All the 46 pharmacists completed the questionnaire and the majority of respondents were male (*N* = 30, 65.2%), in the 23-28 years old range (*N* = 26, 56.5%), at least B.Pharm degree holders (*N* = 25, 54.3%), and have 5-16 years work experience (*N* = 24, 52.2%). Nearly one-third (*N* = 15, 32.6%) of them were members of a certain unspecified professional organization. More than half (*N* = 26, 56.5%) of respondents reported that they are unaware of the CPD concept Table 1.

**Table 1:** Demographic and additional characteristics of the respondents (*N* = 46)

Variables		N (%)
Sex	Female	16 (34.8%)
	Male	30 (65.2%)
Age 30.5 ± 6.9 (mean ± SD)	23–28 year	26 (56.5%)
	29–51 year	20 (43.5%)
Educational level	Diploma	20 (43.5%)
	BPharm degree	25(54.3%)
	MSc degree	1 (2.2%)
Work experience in community pharmacy (years) 5.6 ± 3.9 (mean ± SD)	1–4 year	22 (47.8%)
	5–16 year	24 (52.2%)
Additional work experience	Yes	18 (39.1%)
	No	28 (60.9%)
Employment status	Owner	18 (39.1%)
	Employee	28 (60.9%)
Membership to any professional organization	Yes	15 (32.6%)
	No	31 (67.4%)
Awareness of CPD concept	Yes	20 (43.5%)
	No	26 (56.5%)

**Fig. 1:**
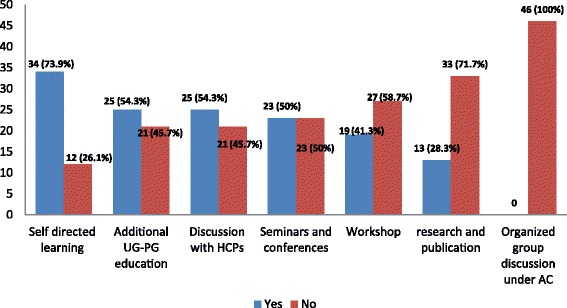
Pattern of CPD engagement among community pharmacists Self directed learning eg reading journal articles, online learning; UG-PG: undergraduate postgraduate; HCPs: health care professionals; AC: accredited coordinator

The respondents reported that they have been engaged in CPD. The main types of activities were self directed leanings (*N* = 34, 73.9%), additional undergraduate or postgraduate education (*N* = 25, 54.3%) as well as discussions with fellow and other health care professionals (N = 25, 54.3%). However they are less involved in research and publication (*N* = 13, 28.3%) and never involved in organized group discussion under accredited coordinator (*N* = 46, 100%) Fig. 1. Females were more engaged than males in research and publication (*X*^*2*^ = 9.480, *p* = 0.005). The diploma holders are more exposed to workshops (*X*^*2*^ = 3.880, *p* = 0.049) and are more involved in research and publication (*X*^*2*^ = 5.820, *p* = 0.016) than their counter groups. Members of professional organizations participate more on seminars and conferences (*X*^*2*^ = 4.847, *p* = 0.028) and are more likely to have discussionswith fellow and other health care professionals (*X*^*2*^ = 5.903, *p* = 0.015). Females think that their previous CPD is enough for preparing them for practice developments (*X*^*2*^ = 14.582, *p* < 0.001) compared to males. No significant group difference was observed in terms of hours spent per week on CPD activity.

The mean hours spent per week on CPD is 4.1 ± 4.0 with minimum of 0 h and maximum of 24 h. Most of the respondents (*N* = 36, 78.3%) agreed that they are not sufficiently engaged on CPD but only 15 (32.6%) reported that their previous CPD is not enough for preparing them for practice developments. More than half (*N* = 26, 56.5%) of respondents reported a receipt of support to engage on CPD.

As shown in Table 2, majority of respondents agree/strongly agree that CPD improves professional practice (*N* = 43, 93.5%) and enhances the status of the profession (N = 43, 93.5%). Thirty nine (84.8%) respondents agree/strongly agree to engage more on CPD and suggested that it should be mandatory. They also seek help in identifying learning needs in CPD. Group based differences on attitude were found only for the sex of respondents as well as their educational level. Males were more likely to accept CPD as enhancing the status of the profession with the public compared to females (females: *N* = 16 mean rank = 18.88; males: *N* = 30, mean rank = 25.97; U = 166, *P* = 0.043). They are also more interested on engaging in CPD activities (females: N = 16 mean rank = 18.53; males: N = 30, mean rank = 26.15; U = 160.5, *p* = 0.044). Those with at least BPharm degree demand additional help to identify their learning needs compared to the diploma holders (diploma holders: *N* = 20, mean rank = 19.10; at least BPharm degree holders: *N* = 26, mean rank = 26.88; U = 171.5, *p* = 0.024). Similarly they are more interested to engage on CPD activity (diploma holders: N = 20, mean rank = 19.08; at least BPharm degree holders: N = 26, mean rank = 26.90; U = 172, *p* = 0.032).

**Table 2:** Attitude of the respondents to CPD (N = 46)

Variable	Response			
	Strongly disagree	Disagree	Agree	Strongly agree
CPD is essential to improve my professional practice	0	3 (6.5)	20 (43.5)	23 (50.0)
CPD enhances status of the profession with other health care professionals	2 (4.3)	1 (2.2)	27 (58.7)	16 (34.8)
CPD enhances status of the profession with the public	0	5 (10.9)	30 (65.2)	11 (23.9)
I want to engage more on CPD	1 (2.2)	6 (13)	23(50.0)	16 (34.8)
CPD should be mandatory	2 (4.3)	5 (10.9)	28 (60.9)	11 (23.9)
I need some help in the process of identifying my learning needs	0	7 (15.2)	28 (60.9)	11 (23.9)

**Fig. 2:**
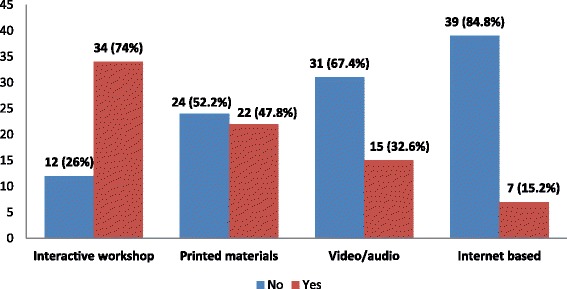
Preference of CPD modes (N = 46) Interactive workshops are the most preferred mode of CPD while internet based approaches were the least ones

Interactive work shop is the most preferred (*N* = 34, 74%) mode of CPD whereas internet based CPD is the least preferred one (7, 15.2%) Fig. 2. Those more educated (*X*^*2*^ = 10.914, *p* = 0.001) and more experienced (*X*^*2*^ = 4.804, *p* = 0.028) were more likely to prefer interactive workshops than their counter groups. Males (*X*^*2*^ = 8.312, *P* = 0.004), older aged groups (28–51 years old) (*X*^*2*^ = 6.972, *p* = 0.008), and the more experienced (5–16 years) (*X*^*2*^ = 4.330, *p* = 0.037) prefer printed materials than their counter groups. Males also prefer video/audio related CPD activity (*X*^*2*^ = 4.514, *p* = 0.034) more than the females.

The top four barriers limiting the community pharmacists'engagement on CPD are lack of relevant learning opportunities (*N* = 36, 78.3%), inaccessibility (location/distance) of group learning activities (*N* = 34, 73.9%), lack of time (*N* = 33, 71.7%) and insufficient resources (*N* = 30, 65.2%) Table 3. The diploma holders consider higher cost as barrier to participation on CPD than their counter groups (*X*^*2*^ = 4.506, *p* = 0.034). Family constraint was more cited as a barrier to CPD engagement by the females (*X*^*2*^ = 4.546, *p* = 0.033), more educated (*X*^*2*^ = 6.002 *P* = 0.014) and pharmacy owners (*X*^*2*^ = 4.785, *p* = 0.029) than their counter groups.

**Table 3:** Perceived barriers to CPD (N = 46)

Variables	Response (*N* = 46)	
	No	Yes
Lack of relevant learning opportunities	10 (21.7%)	36 (78.3%)
Inaccessibility (location/distance) of group learning activities	12 (26.1%)	34 (73.9%)
Lack of time	13 (28.3%)	33 (71.7%)
Insufficient resources to achieve my CPD goals	16 (34.8%)	30 (65.2%)
Job constraints	18 39.1%)	28 (60.9%)
Cost Higher cost of participation	20 (43.5%)	26 (56.5%)
Low personal priority of learning in relation to other activities	22 (47.8%)	24 (52.2%)
Lack of quality learning activities	23 (50%)	23 (50%)
Family constraints (e.g. spouse, children)	27 (58.7%)	19 (41.3%)
Uninteresting subjects or topics	28 (60.9%)	18 (39.1%)

As shown in Table 4, there are no group differences with respect to self reported awareness on CPD except for membership of professional organizations. Thus members are 4.9 times more likely to believe that they are aware of CPD compared to those who are not members (*p* = 0.027).

**Table 4:** Pearson’s Chi-square test of independence on self reported awareness on CPD concept (N = 46)

Variables		I am aware of CPD concept (*N* = 20)
Sex	Female 8 (50%)	*X*^*2*^ = 0.425, df = 1, *p* = 0.515
	Male 12 (40%)	
Age	23–28 year 11 (42.3%)	*X*^*2=*^0.033, df = 1, *p* = 0.855
	29–51 year 9 (45%)	
Educational level	Diploma 9 (45%)	*X*^*2*^ = 0.033, df = 1, *p* = 0.855
	At least BPharm 11 (42.3%)	
Work experience in community pharmacy (years)	1–4 year 10 (45%)	*X*^*2*^ = 0.067, df = 1, *p* = 0.796
	5–16 year 10 (47.4%)	
Additional work experience	Yes 11 (61.1%)	*X*^*2*^ = 3.741, df = 1, *p* = 0.053
	No 9 (32.1%)	
Employment status	Owner 10 (55.6%)	*X*^*2*^ = 1.755, df = 1, *p* = 0.185
	Employee 10 (35.7%)	
Membership to any professional organization	Yes 10 (66.7%)	*X*^*2*^ = 4.870, df = 1, *p* = 0.027^a^
	No 10 (32.3%)	

^a^ = statistically significant at *p* < 0.05

## Discussion

A 2014 global report on CPD/CE in pharmacy stated that Ethiopia is one of those countries which did not implemented or used recommendations of the 2002 FIP Statement on CPD [11]. However, the Ethiopian Pharmaceutical Association (EPA), established in 1974, is now striving to find ways of improving the training and utilization of pharmacy professionals as one of its objectives. It has conducted CPD needs assessment and is on track to provide CPD to pharmacists [12]. Thus membership to such professional organization creates the platform to improve professional practice of pharmacists. In this regard, it is encouraging that nearly one-third of respondents in the present study were members of a certain professional organization. The FIP definition of CPD [1] was stated by the interviewer before proceeding to the other questions. Majority of respondents reported that they are unaware of the CPD concept. Pharmacists in developing countries, unless exposed to CPD, are less likely to be aware of its concepts. A study done in India on 48 pharmacists reported that none of the participants were aware of CPD concept prior to a CPD intervention [13]. Despite self reported lack of awareness, the respondents in the present study are engaged in CPD activities. The main modes were self directed leanings, additional undergraduate or postgraduate education as well as having discussions with fellow and other health care professionals. However, less involvement is reported in research and publication and organized group discussion under accredited coordinator. Being a relatively new idea [1], CPD seems at its infancy in Ethiopia and thus involvement of community pharmacists is largely in the form of the traditional CE approach. Females (*p* = 0.005) and diploma holders (*p* = 0.016) were more involved in research and publication than the counter groups. Membership to a professional organization increased participation on seminars and conferences (*p* = 0.028) and discussions with fellow and other health care professionals (*p* = 0.015). Compared to males, female pharmacists think that their previous CPD activity is enough for preparing them for practice developments (*p* < 0.001). Excluding the continuing undergraduate or post graduate education, the mean hours spent per week on CPD was 4.1 ± 4.0 with a minimum of 0 h and maximum of 24 h. No significant group difference was observed in terms of hours per week spent on CPD activity. In line with this, most of the respondents agreed that they are not sufficiently engaged on CPD. It might be associated with lack of support to engage on CPD as stated by some respondents. In general most believe CPD improves professional practice similar to that reported by Power et al. [14]. They are interested to engage more on CPD and opined making CPD mandatory. However they demand some help in identifying learning needs in CPD similar to previous studies [14–16]. Tijin et al. also reported that motivation of pharmacists has influence on CE practice [5].

Compared to females, males were more likely to be interested on engaging in CPD activities (*p* = 0.044). Those more educated i.e. with at least BPharm degree are more interested to engage CPD (*p* = 0.032) and demand additional help to identify their learning needs compared to the less educated i.e. diploma holders (*p* = 0.024). The reasons however need to be further investigated.

Preference to the type of CPD differs among subgroups of community pharmacists. Interactive work shop is the most preferred type of CPD whereas internet based CPD is least preferred one. Similar preference to interactive workshop is also reported from previous studies [3, 17]. Those more educated (*p* = 0.001) and more experienced (*p* = 0.028) are more likely to prefer interactive workshops than their counter groups. Printed materials are also more preferred by males (*P* = 0.004), older aged groups i.e. 28–51 years old (*p* = 0.008), and the more experienced i.e. 5–16 years (*p* = 0.037) than their counter groups. Males also prefer video/audio related CPD activity (*p* = 0.034) more than females. These findings will help in desgning CPD modes for community pharmacists.

The top four barriers limiting the community pharmacist’s engagement on CPD were lack of relevant learning opportunities, inaccessibility (location/distance) of group learning activities, and lack of time and insufficient resources to achieve CPD goals. These are also reported in previous studies [12, 18]. The diploma holders identified cost as barrier to participation on CPD than their counter groups (p = 0.034). Females (*p* = 0.033), more educated (*p* = 0.014) and owners (*p* = 0.029) cited family constraint as a barrier to CPD engagement more than their counter groups. Members of a professional organization are 4.9 times more likely to believe they are aware of CPD compared to non-members (*p* = 0.027). These findings shall be emphasized in implementing CPD for community pharmacists in Ethiopia.

With an ever increasing expenditure on pharmaceuticals [19], CPD by pharmacists would be an important means of efficient resource utilization.

### Limitation of the study

The main limitation of the present study is small sample size and that involvement in CPD was measured by self report of respondents. This may inflate some of the findings [5]. Other methods should be considered in further studies.

## Conclusion

The community pharmacists in Gondar, Northwest Ethiopia lack awareness of CPD concept. However they are engaged in CPD, demonstrated good attitude, and demand more support. The main barrier is lack of opportunities related to CPD.

## Abbreviation

CPD: Continuous professional development
